# Optic Disc - Fovea Distance, Axial Length and Parapapillary Zones. The Beijing Eye Study 2011

**DOI:** 10.1371/journal.pone.0138701

**Published:** 2015-09-21

**Authors:** Rahul Arvo Jonas, Ya Xing Wang, Hua Yang, Jian Jun Li, Liang Xu, Songhomitra Panda-Jonas, Jost Bruno Jonas

**Affiliations:** 1 Beijing Institute of Ophthalmology, Beijing Tongren Eye Center, Beijing Tongren Hospital, Capital Medical University, Beijing Ophthalmology and Visual Science Key Lab, Beijing, China; 2 Department of Ophthalmology, Medical Faculty Mannheim of the Ruprecht-Karls-University of Heidelberg, Mannheim, Germany; University of Houston, UNITED STATES

## Abstract

**Purpose:**

To measure the distance between the optic disc center and the fovea (DFD) and to assess its associations.

**Methods:**

The population-based cross-sectional Beijing Eye Study 2011 included 3468 individuals aged 50+ years. The DFD was measured on fundus photographs.

**Results:**

Readable fundus photographs were available for 2836 (81.8%) individuals. Mean DFD was 4.76 ± 0.34mm (median: 4.74 mm; range: 3.76–6.53mm). In multivariate analysis, longer DFD was associated with longer axial length (*P*<0.001; standardized correlation coefficient beta: 0.62), higher prevalence of axially high myopia (*P*<0.001; beta:0.06), shallower anterior chamber depth (*P*<0.001; beta:-0.18), thinner lens thickness (*P* = 0.004; beta: -0.06), smaller optic disc-fovea angle (*P* = 0.02; beta: -0.04), larger parapapillary alpha zone (*P* = 0.008; beta: 0.05), larger parapapillary beta/gamma zone (*P*<0.001; beta: 0.11), larger optic disc area (*P*<0.001; beta: 0.08), lower degree of cortical cataract (*P* = 0.002; beta: -0.08), and lower prevalence of age-related macular degeneration (*P* = 0.001; beta: -0.06). Bruch´s membrane opening-fovea distance (DFD minus disc radius minus parapapillary beta/gamma zone width) in non-glaucomatous eyes was not significantly (*P* = 0.60) related with axial length in emmetropic or axially myopic eyes (axial length ≥23.5 mm), while it increased significantly (*P*<0.001; r: 0.32) with longer axial length in eyes with an axial length of <23.5mm. Ratio of mean DFD to disc diameter was 2.65 ± 0.30. If the ratio of disc-fovea distance to disc diameter was considered constant and if the individual disc diameter was calculated as the individual disc-fovea distance divided by the constant factor of 2.65, the resulting calculated disc diameter differed from the directly measured disc diameter by 0.16 ±0.13 mm (median: 0.13 mm, range: 0.00–0.89 mm) or 8.9 ± 7.3% (median: 7.4%; range: 0.00–70%) of the measured disc diameter.

**Conclusions:**

DFD (mean: 4.76mm) increases with longer axial length, larger parapapillary alpha zone and parapapillary beta/gamma zone, and larger disc area. The axial elongation associated increase in DFD was due to an enlargement of parapapillary beta/gamma zone while the Bruch’s membrane opening-fovea distance did not enlarge with longer axial length. This finding may be of interest for the process of emmetropization and myopization. Due to its variability, the disc-fovea distance has only limited clinical value as a relative size unit for structures at the posterior pole.

## Introduction

The optic disc-fovea distance is an anatomical landmark of the posterior fundus and has not yet extensively been examined [1–9]. It has previously been used to estimate the optic disc diameter or the size of other structures located in the posterior ocular segment [1–4, 6,9]. The disc-fovea distance may be of basic importance as an anatomical measure of the posterior fundus, and it may be associated with other anatomical parameters and with the prevalence of ocular abnormalities and disorders. One may postulate that a postnatally enlarged disc-fovea distance can be associated with a stretching of the posterior fundus and thus with an increased inter-photoreceptor distance, through which it may have a direct effect on visual acuity. The disc-fovea distance can ophthalmoscopically be estimated without using sophisticated imaging tools and may thus have importance in the daily routine examination of patients. Since studies which systematically assessed the disc-fovea distance have been lacking so far, we conducted this population-based study to measure the disc-fovea distance and to correlate it with other ocular and systemic parameters.

## Methods

The Beijing Eye Study 2011 is a population-based cross-sectional study in Northern China. It has been described in detail previously [10–12]. The Medical Ethics Committee of the Beijing Tongren Hospital approved the study protocol and all participants gave informed written consent. Inclusion criterion was an age of 50+ years. In the survey, 3468 individuals (1963 (56.6%) women) participated out of 4403 eligible individuals (response rate: 78.8%). There were 1633 (47.1%) subjects (943 (57.7%) women) from the rural region and 1835 (52.9%) subjects (1020 (55.6%) women)) for the urban region. The mean age was 64.6 ± 9.8 years (median, 64 years; range, 50–93 years).

**Fig 1:**
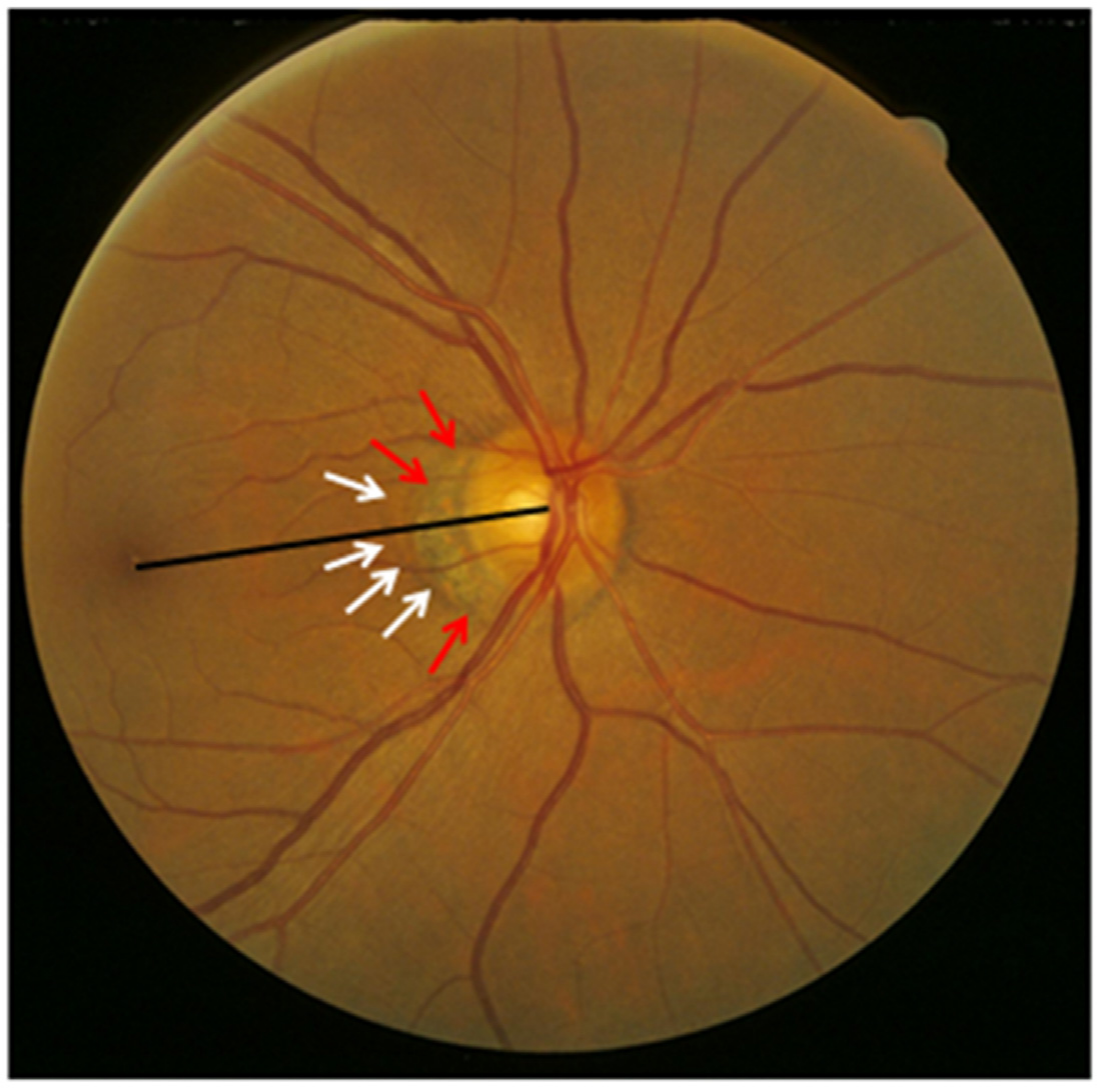
Fundus photograph showing the optic disc—fovea line (black line), outer border of parapapillary alpha zone (white arrows) and the outer border of parapapillary beta (gamma) zone (red arrows).

All participants underwent a structured questionnaire by trained health staff with standardized questions on their family status, level of education, income, quality of life, psychic depression, physical activity, and known major systemic diseases, systemic examinations, and a comprehensive ophthalmic examination. The examinations were performed in the communities, either in schoolhouses or in community houses. Using a stadiometer, we measured body height in a standardized manner with the shoes routinely removed. Body weight and the circumferences of the waist and hip were determined. The ocular examinations included assessment of presenting visual acuity and uncorrected visual acuity. If uncorrected visual acuity was lower than 1.0 (or logMAR (negative decadal logarithm of the minimal angle of resolution) higher than 0.00), best corrected visual acuity was assessed after automatic refractometry (Auto Refractometer AR-610, Nidek Co., Ltd, Tokyo, Japan) was carried out. Intraocular pressure was determined by pneumotonometry. A slit lamp examination carried out by an experienced ophthalmologist assessed lid abnormalities, Meibomian gland dysfunction, corneal disorders, and peripheral anterior chamber depth using van Herick’s method. Gonioscopy was routinely performed for all study participants. Applying optical low-coherence reflectometry (Lensstar 900® Optical Biometer, Haag-Streit, 3098 Koeniz, Switzerland), we carried out an ocular biometry of the right eyes for assessment of the anterior corneal curvature, central corneal thickness, anterior chamber depth, lens thickness and axial length. Five measurements were performed, and the mean value was taken for further statistical analysis. After medical dilatation of the pupil (tropicamide 1% eye drops; Mydrin; Santen; Japan), a second slit lamp assisted biomicroscopy searched for pseudoexfoliation syndrome. Digital photographs of the cornea and lens and retro-illuminated photographs of the lens were taken using the Neitz CT-R camera (Neitz Instruments Co., Tokyo, Japan). Monoscopic photographs of the macula and optic disc were taken using a fundus camera (Type CR6-45NM, Canon Inc. U.S.A.). The degree of cataract was determined using the standardized lens photographs as described recently [13]. Diabetic retinopathy was diagnosed on the fundus photographs [14]. Using the fundus photographs, we measured the distance between the optic disc center and the foveola and the angle between the disc-fovea line and the horizontal (Fig 1). The center of the disc was assessed as the middle point of the line connecting the middle point of the minimal disc diameter and the middle point of maximal disc diameter. The location of the foveola was determined as the location of the foveal reflex in eyes with a foveal reflex, as the center of the macula wall reflex in eyes showing a macular wall reflex but no foveal reflex, and as the center of the apparently avascular zone in those eyes which did not present any macular reflexes. The location of the foveola as determined on the fundus photographs was controlled by images of the macula obtained by spectral domain optical coherence tomography (SD-OCT; Spectralis®, Wavelength: 870nm; Heidelberg Engineering Co., Heidelberg, Germany). Seven sections, each comprising 100 averaged scans, were obtained in a rectangle measuring 5° x 30° and which was centered onto the fovea. On these OCT images, the foveola was detectable as the foveal depression of the macular thickness profile. Using the optic disc photographs, we determined the width of parapapillary beta / gamma zone defined as the parapapillary zone with visible sclera and visible large choroidal vessels [15]. If beta / gamma zone was present, it was located at the peripapillary ring of the optic nerve head. It usually was separated from the remaining retina by a parapapillary alpha zone. The latter showed an irregular hyperpigmentation and hypopigmentation (Fig 1). Using the same disc photographs, we had previously measured the area and diameter of the optic nerve heads [16]. To obtain the disc-fovea distance and the beta / gamma zone measurements in real size units, i.e. to correct for the lateral magnification of the fundus image, we used the Littmann-Bennett method to correct the image magnification which was caused by the optic media of the eye and by the fundus camera [17–19]. For the latter, we used the axial length as the basic principle ocular parameter for the calculations. We additionally determined a parameter called Bruch´s membrane opening (BMO)-to-fovea distance as the difference of the optic disc center-to-fovea distance minus the optic disc radius minus the parapapillary beta / gamma zone width.

Statistical analysis was performed using a commercially available statistical software package (SPSS for Windows, version 22.0, IBM-SPSS, Chicago, IL, USA). Inclusion criteria for the study were the availability of measurements of the disc-fovea distance and the availability of axial length measurements. Only the right eye per individual was taken for the statistical analysis. In a first step, we examined the distribution of the disc-fovea distance using the Kolmogorov-Smirnov test. We then calculated the mean ± standard deviations of the parameter. In a second step of the analysis, we performed a univariate analysis of the associations between the disc-fovea distance and other ocular and systemic variables. In a third step, we conducted a multivariate regression analysis, with the disc-fovea distance as dependent variable and all those parameters as independent variables which were significantly associated with the disc-fovea parameters in the univariate analysis. From the list of independent parameters we then dropped step by step those parameters which were no longer significantly associated. 95% confidence intervals (CI) were presented. All *P*-values were two-sided and were considered statistically significant if the values were smaller than 0.05.

## Results

Out of the 3468 subjects, readable fundus photographs for the measurement of the disc-fovea distance and axial length measurements were available for 2836 (81.8%) individuals with a mean age of 63.3 ± 9.3 years (range: 50 to 91 years) and a mean axial length of 23.2 ± 1.0 mm (range: 18.96–28.87 mm). The individuals excluded from the study as compared with those included were significantly older (70.5 ± 9.7 years versus 63.3 ± 9.3 years; *P*<0.001), had a longer axial length (23.8 ± 1.9 mm versus 23.2 ± 1.0 mm; *P*<0.001), and were significantly more myopic (-1.29 ± 3.67 diopters versus -0.04 ± 1.65 diopters; *P*<0.001). Both groups did not vary significantly in gender (*P* = 0.33).

**Fig 2:**
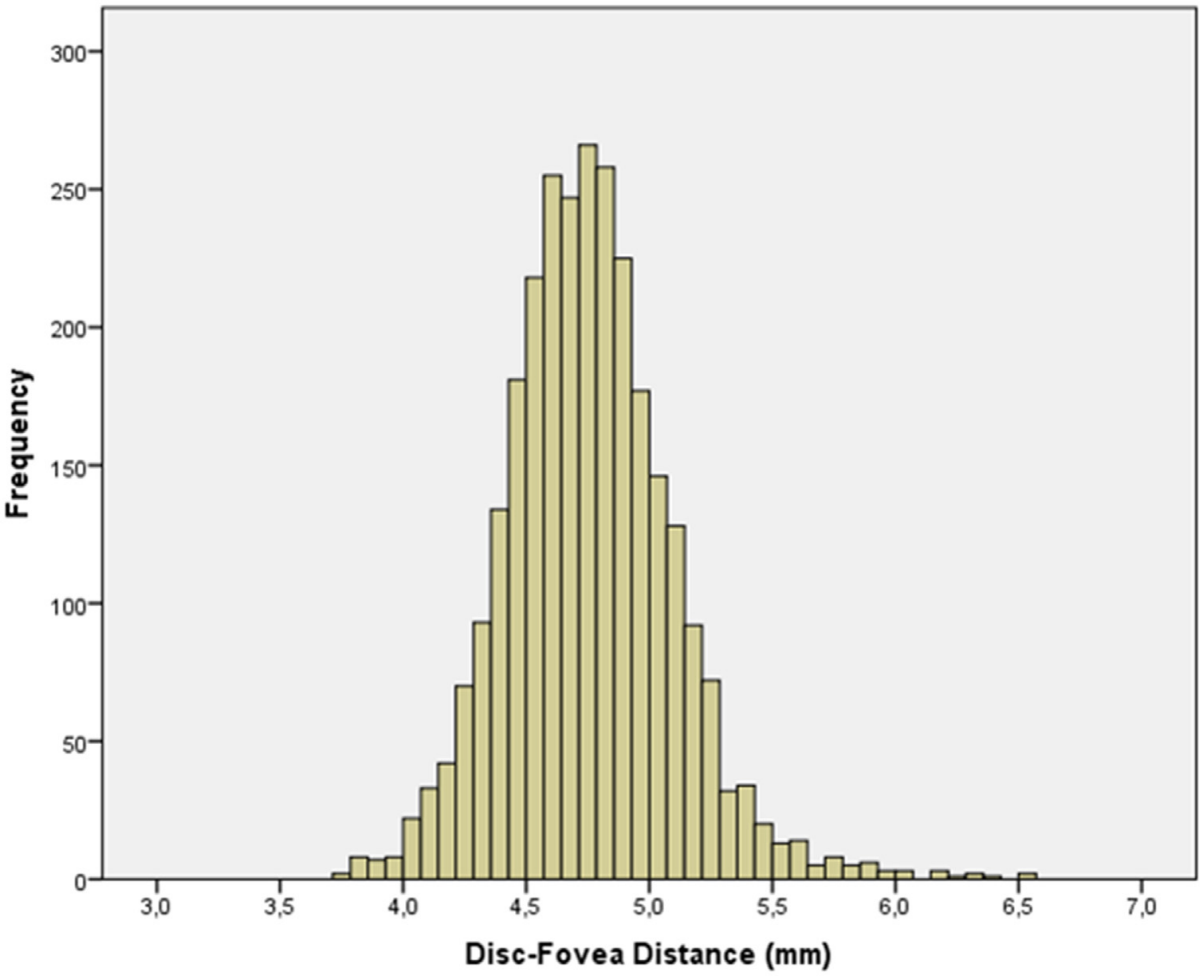
Distribution of the Disc-Fovea Distance.

**Fig 3:**
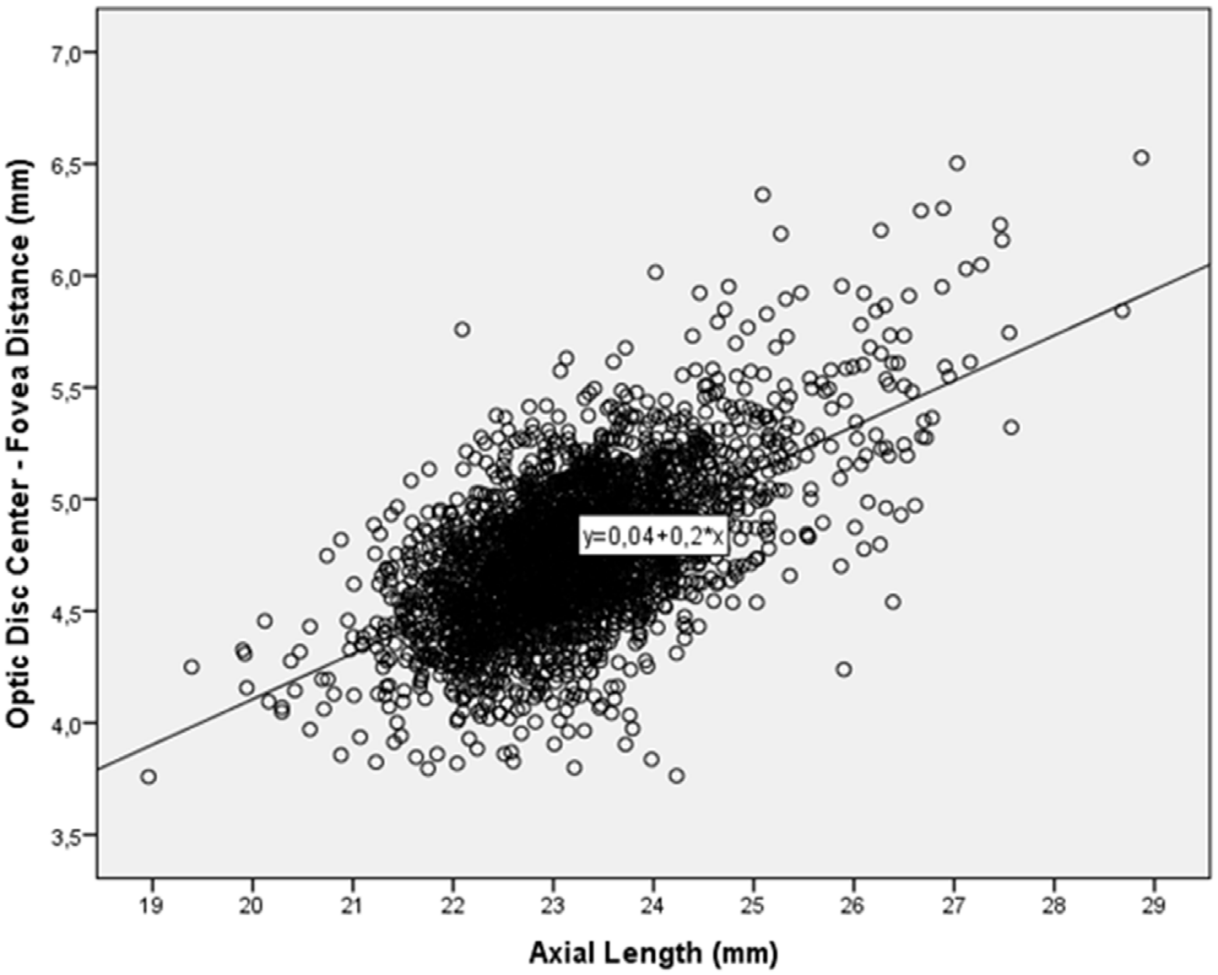
Association between Axial Length and Optic Disc-Fovea Distance.

Mean disc-fovea distance was 4.76 ± 0.34 mm (median: 4.74 mm; range: 3.76–6.53 mm). It was not normally distributed (*P*<0.001) (Fig 2). In univariate analysis, longer disc-fovea distance was significantly associated with the systemic parameters of male gender (*P*<0.001; correlation coefficient r: -0.12), urban region of habitation (*P*<0.001, r: 0.13), higher level of education (*P*<0.001; r: 0.20), higher body height (*P*<0.001; r: 0.23), heavier body weight (*P*<0.001; r: 0.08), lower systolic blood pressure (*P* = 0.001; r: -0.08), lower blood concentrations of high-density lipoproteins (*P* = 0.01, r: -0.04) and lower blood concentration of glucose (*P* = 0.01; r: -0.06). Longer disc-fovea distance was associated (univariate analysis) with the ocular parameters of longer axial length (*P*<0.001; r: 0.20) (Fig 3), thicker central corneal thickness (*P*<0.001; r: 0.001), deeper anterior chamber depth (*P*<0.001; r: 0.11), larger anterior corneal curvature radius (*P*<0.001; r: 0.38), thinner lens thickness (*P*<0.001; r: -0.07), more myopic refractive error (*P*<0.001; r: -0.08), smaller disc-fovea angle (*P* = 0.002; r: -0.006), larger parapapillary alpha zone (*P*<0.001; r: 0.07), larger parapapillary beta/gamma zone (*P*<0.001; r: 0.11), larger optic disc area (*P*<0.001; r: 0.07), lower prevalence of early age-related macular degeneration (*P* = 0.03; r: -0.04), intermediate age-related macular degeneration (*P* = 0.001; r: -0.07) and of any age-related macular degeneration (*P* = 0.001; r: -0.07), lower degree of cortical cataract (*P*<0.001; r: -0.08), higher prevalence of open-angle glaucoma (*P* = 0.001; r: 0.07), higher intraocular pressure (*P* = 0.03; r: 0.04) and higher prevalence of high axial myopia (defined as axial length ≥26.5 mm) (*P*<0.001; r: 0.28). The disc-fovea distance was not significantly associated with age (*P* = 0.89), body mass index (*P* = 0.11), diastolic blood pressure (*P* = 0.09), blood concentration of low-density lipoproteins (*P* = 0.13), cholesterol (*P* = 0.11), and triglycerides (*P* = 0.56), degree of nuclear cataract (*P* = 0.29) and subcapsular posterior cataract (*P* = 0.10), and prevalence of angle-closure glaucoma (*P* = 0.86), diabetic retinopathy (*P* = 0.16) and retinal vein occlusions (*P* = 0.60).

For the multivariate analysis with the disc-fovea distance as dependent variable we included all those parameters as independent variables which were significantly associated with the disc-fovea distance in the univariate analysis. Due to collinearity, we dropped prevalence of early and intermediate age-related macular degeneration (due to collinearity with any age-related macular degeneration), and refractive error due to the collinearity with axial length. Since they were no longer significantly associated with disc-fovea distance, we then deleted from the list of independent parameters the anterior corneal curvature radius (*P* = 0.95), sex (*P* = 0.66), blood concentration of glucose (*P* = 0.85), body weight (*P* = 0.66), intraocular pressure (*P* = 0.56), central corneal thickness (*P* = 0.44), prevalence of open-angle glaucoma (*P* = 0.43), region of habitation (*P* = 0.41), level of education (*P* = 0.38), systolic blood pressure (*P* = 0.27), blood concentration of high-density lipoproteins (*P* = 0.24) and body height (*P* = 0.40). In the final model (overall correlation coefficient r: 0.64), longer disc-fovea distance was significantly associated with longer axial length (*P*<0.001), higher prevalence of axially high myopia (*P*<0.001), shallower anterior chamber depth (*P*<0.001), thinner lens thickness (*P* = 0.004), smaller optic disc-fovea angle (*P* = 0.02), larger parapapillary alpha zone (*P* = 0.008), larger parapapillary beta/gamma zone (*P*<0.001), larger optic disc area (*P*<0.001), lower degree of cortical cataract (*P* = 0.002), and lower prevalence of age-related macular degeneration (*P* = 0.001) (Table 1).

**Table 1:** Associations (Multivariate Analysis) between the Disc-Fovea-Distance and Ocular and Systemic Parameters in the Beijing Eye Study 2011.

Parameter	*P*-Value	Standardized Correlation Beta	Non-Standardized Correlation Coefficient B	95% Confidence Interval	Variance Inflation Factor
Axial Length (mm)	<0.001	0.62	0.21	0.20, 0.23	1.69
Axial High Myopia (Axial Length ≥26.5 mm)	0.002	0.06	0.27	0.10, 0.44	1.12
Anterior Chamber Depth (mm)	<0.001	-0.18	-0.18	-0.23, -0.13	1.96
Lens Thickness (mm)	0.004	-0.06	-0.07	-0.11, -0.02	1.41
Optic Disc-Fovea Angle	0.02	-0.04	-0.004	-0.008, -0.001	1.01
Parapapillary Alpha Zone Area (mm^2^)	0.008	0.05	0.03	0.01, 0.05	1.05
Parapapillary Beta/Gamma Zone Area (mm^2^)	<0.001	0.11	0.05	0.03, 0.06	1.11
Optic Disc Area (mm^2^)	<0.001	0.08	0.06	0.03, 0.08	1.06
Cortical Cataract	<0.001	-0.08	-0.22	-0.32, -0.12	1.02
Any Age-Related Macular Degeneration	0.001	-0.06	-0.05	-0.07, -0.02	1.01

The ratio of the mean disc-fovea distance to optic disc diameter was 2.65 ± 0.30. If one assumed that the ratio of disc-fovea distance to disc diameter was constant between individuals and if the individual disc diameter was calculated as the individual disc-fovea distance divided by the constant factor of 2.65, the resulting calculated disc diameter differed from the directly measured disc diameter by a mean amount of 0.16 ±0.13 mm (median: 0.13 mm, range: 0.00–0.89 mm) or 8.9 ± 7.3% (median: 7.4%; range: 0.00–70%) of the measured disc diameter.

**Fig 4:**
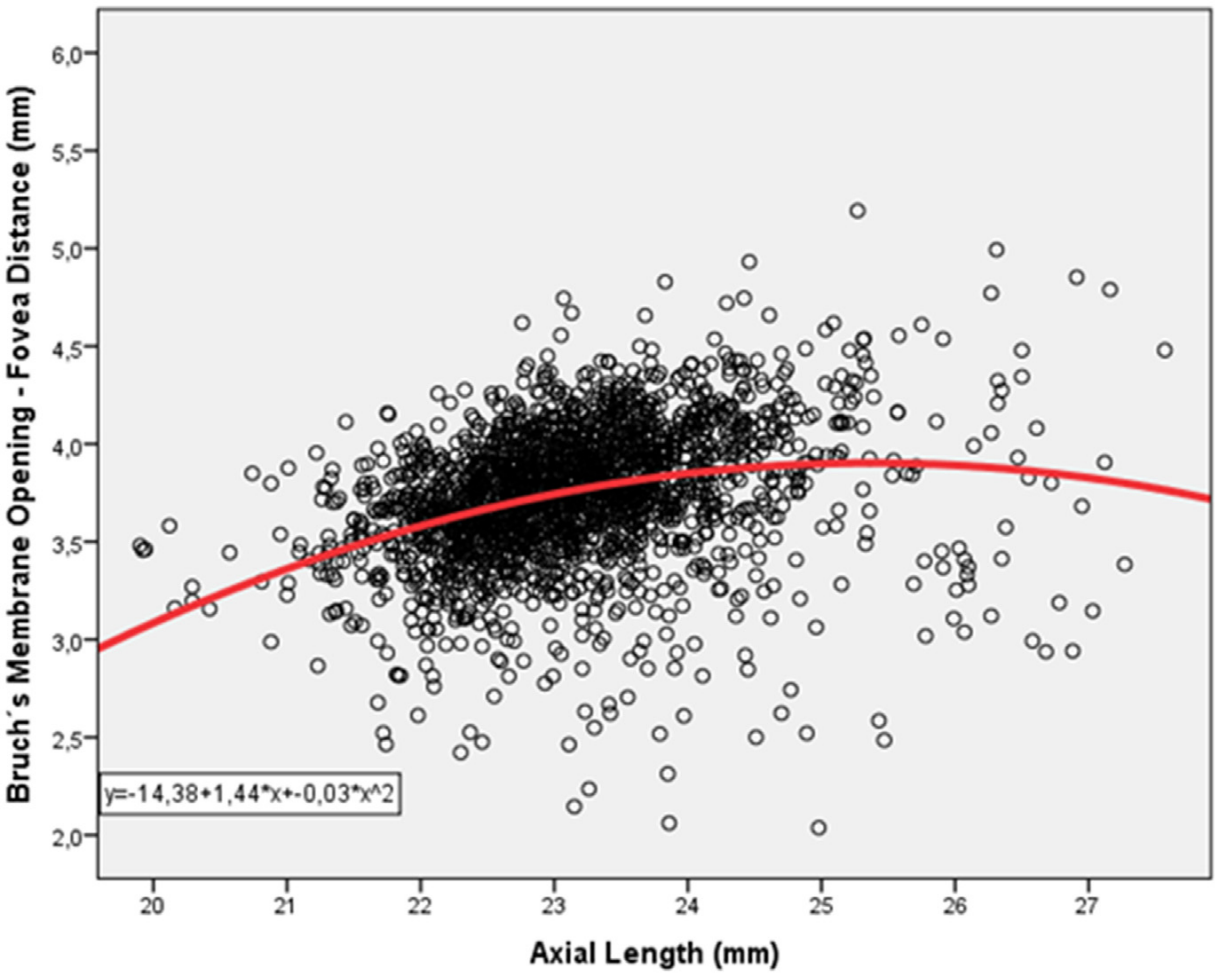
Distribution of Axial Length and Bruch´s Membrane Opening—Fovea Distance in Non-Glaucomatous Individuals.

After excluding eyes with glaucomatous optic neuropathy, mean BMO-fovea distance was 3.74 ± 0.36 mm (median: 3.75 mm; range: 2.04; 5.19 mm). Mean BMO-fovea distance significantly increased with longer axial length (*P*<0.001; r: 0.28). Within the subgroup of eyes with an axial length of shorter than 23.5 mm, BMO-fovea distance increased with longer axial length (*P*<0.001; r: 0.32), while in the subgroup with an axial length of ≥23.5 mm, BMO-fovea distance was not associated with axial length (*P* = 0.60) (Fig 4). In the same subgroup of individuals with an axial length of ≥23.5 mm, the disc-fovea distance significantly increased with longer axial length (*P*<0.001; standardized correlation coefficient beta: 0.56; correlation coefficient B: 0.25; 95%CI: 0.22, 0.27) (Fig 3). The cut-off value of 23.5 mm was chosen since it appeared on the scattergram that at that value the situation of a positive relationship between longer axial length and longer BMO-fovea distance changed to the situation of a lack of such an association (Fig 4).

Within the subgroup with an axial length of <23.5 mm (n = 1896 individuals), BMO-fovea distance was significantly (univariate analysis) associated with male gender (*P*<0.001; r: -0.12), younger age (*P*<0.001, r: -0.12), rural region of habitation (*P* = 0.007, r: -0.07), higher body height (*P*<0.001; r: 0.17), heavier body weight (*P*<0.001; r: 0.10), longer axial length (*P*<0.001; r: 0.32) (Fig 4), larger anterior corneal curvature radius (*P*<0.001; r: 0.25), more myopic refractive error (*P*<0.001; r: -0.13), larger parapapillary alpha zone (*P*<0.001; r: 0.11), smaller parapapillary beta/gamma zone (*P*<0.001; r: -0.53), and smaller optic disc area (*P*<0.001; r: -0.23), and lower prevalence of early age-related macular degeneration (*P* = 0.049; r: -0.05), and lower degree of cortical cataract (*P*<0.001; r: -0.13). The BMO-fovea distance was not significantly associated with level of education (*P* = 0.08), central corneal thickness (*P* = 0.49), anterior chamber depth (*P* = 0.051), lens thickness (*P* = 0.14), disc-fovea angle (*P* = 0.57), and prevalence of intermediate age-related macular degeneration (*P* = 0.17), and of late age-related macular degeneration (*P* = 0.94). In the multivariate analysis, we first dropped refractive error due to reasons of collinearity with axial length. We then deleted the parameters of body weight (*P* = 0.51), region of habitation (*P* = 0.51), and body height (*P* = 0.38). In the final model, longer BMO-fovea distance was associated with younger age (*P* = 0.02), male gender (*P* = 0.003), longer axial length (*P*<0.001), longer anterior corneal curvature radius (*P*<0.001), larger parapapillary alpha zone (*P*<0.001), smaller beta/gamma zone (*P*<0.001) and smaller optic disc (*P*<0.001), lower prevalence of early age-related macular degeneration (*P* = 0.03) and lower degree of cortical cataract (*P* = 0.02) (Table 2). In this subgroup with an axial length of <23.5 mm, longer axial length was significantly associated with the disc-fovea distance (*P*<0.001; r: 0.39).

**Table 2:** Associations (Multivariate Analysis) between the Bruch’s Membrane Opening-Fovea-Distance and Ocular and Systemic Parameters in Non-Glaucomatous Individuals with an Axial Length of <23.5 mm in the Beijing Eye Study 2011.

Parameter	*P*-Value	Standardized Correlation Beta	Non-Standardized Correlation Coefficient B	95% Confidence Interval	Variance Inflation Factor
Age (Years)	0.02	-0.06	-0.002	-0.004, 0.000	1.14
Gender	0.003	-0.07	-0.05	-0.08, -0.02	1.08
Axial Length (mm)	<0.001	0.21	0.12	0.09, 0.15	1.49
Anterior Corneal Curvature Radius (mm)	<0.001	0.15	0.22	0.14, 0.30	1.41
Parapapillary Alpha Zone Area (mm^2^)	<0.001	0.09	0.06	0.03, 0.09	1.08
Parapapillary Beta/Gamma Zone Area (mm^2^)	<0.001	-0.46	-0.41	-0.45, -0.37	1.06
Optic Disc Area (mm^2^)	<0.001	-0.17	-0.11	-0.14, -0.08	1.09
Early Age-Related Macular Degeneration	0.03	-0.05	-0.05	-0.10, -0.01	1.01
Cortical Cataract Degree	0.02	-0.06	-0.15	-0.27, -0.03	1.11

Within the subgroup with an axial length of ≥23.5 mm (n = 940 individuals), BMO-fovea distance was significantly (univariate analysis) associated with younger age (*P*<0.001, r: -0.18), larger parapapillary alpha zone (*P* = 0.008; r: 0.10), smaller parapapillary beta/gamma zone (*P*<0.001; r: -0.67), smaller optic disc area (*P*<0.001; r: -0.14), and lower degree of cortical cataract (*P*<0.001; r: -0.13). The BMO-fovea distance was not significantly associated with gender (*P* = 0.51), rural region of habitation (*P* = 0.30), body height (*P* = 0.55), body weight (*P* = 0.18), level of education (*P* = 0.40), axial length (*P* = 0.60) (Fig 4), larger anterior corneal curvature radius (*P* = 0.005; r: 0.11), thinner lens thickness (*P* = 0.001; r: -0.13), central corneal thickness (*P* = 0.14), refractive error (*P* = 0.98), anterior chamber depth (*P* = 0.41), disc-fovea angle (*P* = 0.20), and prevalence of early age-related macular degeneration (*P* = 0.83), intermediate age-related macular degeneration (*P* = 0.39), and of late age-related macular degeneration (*P* = 0.34). In this subgroup with an axial length of ≥23.5 mm, longer axial length was significantly associated with the disc-fovea distance (*P*<0.001; r: 0.57).

In the multivariate analysis, we dropped age (*P* = 0.55) and degree of cortical cataract (*P* = 0.20), so that in the final model, a longer BMO-fovea distance in the subgroup with an axial length of ≥23.5 mm was associated with larger parapapillary alpha zone (*P* = 0.009), smaller beta/gamma zone (*P*<0.001), and smaller optic disc area (*P*<0.001) (Table 3).

**Table 3:** Associations (Multivariate Analysis) between the Bruch’s Membrane Opening-Fovea-Distance and Ocular and Systemic Parameters in Non-Glaucomatous Individuals with an Axial Length of ≥23.5 mm in the Beijing Eye Study 2011.

Parameter	*P*-Value	Standardized Correlation Beta	Non-Standardized Correlation Coefficient B	95% Confidence Interval	Variance Inflation Factor
Parapapillary Alpha Zone Area (mm^2^)	0.009	0.08	0.05	0.01, 0.08	1.01
Parapapillary Beta/Gamma Zone Area (mm^2^)	<0.001	-0.66	-0.37	-0.40, -0.34	1.01
Optic Disc Area (mm^2^)	<0.001	-0.10	-0.09	-0.14, -0.04	1.01

## Discussion

In our population-based study, the optic disc-fovea distance (mean: 4.76 ± 0.34 mm) was significantly associated with longer axial length (*P*<0.001), higher prevalence of axially high myopia (*P*<0.001), shallower anterior chamber depth (*P*<0.001), thinner lens thickness (*P* = 0.004), smaller optic disc-fovea angle (*P* = 0.02), larger parapapillary alpha zone (*P* = 0.008), larger parapapillary beta/gamma zone (*P*<0.001), larger optic disc area (*P*<0.001), lower degree of cortical cataract (*P* = 0.002), and lower prevalence of age-related macular degeneration (*P* = 0.001) (Table 1). If the disc-fovea distance was reduced by the disc radius and by the width of parapapillary beta/gamma zone in non-glaucomatous eyes with an axial length of ≥23.5 mm, the remaining so called BMO-fovea distance was not significantly associated with axial length.

The mean value of the disc-fovea distance of 4.76 mm is in agreement with previous studies. In a study by Knaapi and coworkers, the disc-fovea distance was measured on digital photographs obtained from 27 prematurely born children at an age of 10–11 years [9]. The mean disc-macula distance was 4.74 ± 0.29 mm, a value almost identical with the one obtained in our study. In contrast to our study, Knaapi and associates did not find significant associations between the disc-fovea distance and refractive error (or axial length), although one child with axially high myopia had an above-average disc-fovea distance of 6.35 mm. When comparing the results obtained in Knaapi´s study and the findings of our investigation, one has to take into account the marked difference between both studies in the age and in the background of the study participants. Lee and colleagues measured the distance between the temporal optic disc margin to the fovea distance in 88 patients with normal-tension glaucoma [8]. They found that in patients with a sparing of the central field the disc margin-fovea distance (3.88 ± 0.28 mm) was longer (*P* = 0.002) than in the group with glaucomatous central visual field loss (3.64 ± 0.40 mm) [8]. Interestingly, parapapillary atrophy was wider (*P* = 0.03) in the group with sparing of the central visual field and longer disc margin-fovea distance. It fits with our observation, that a longer disc-fovea distance was significantly associated with larger parapapillary beta/gamma zone in the multivariate analysis in our study population. Van de Put and coworkers measured the disc-fovea distance in 183 diabetic patients without retinopathy [7]. Similar to our study, the mean disc-fovea distance was 4.72 ± 0.27 mm, and the disc-fovea distance decreased by 0.06 mm (*P*<0.001) per diopter increase in spherical equivalent of refractive error. Examining 51 preterm and full-term infants, de Silva and colleagues measured a mean optic disc-fovea distance of 4.4 ± 0.4 mm, without difference between the preterm infants and the full-term infants [6]. This value measured in infants was lower than the value obtained in our adult study participants. Interestingly, the mean ratio of disc-fovea distance to optic disc diameter was higher in the infants than in our study (3.76 versus 2.65).

The association between a longer disc-fovea distance and longer axial length can be explained by the enlargement of the posterior segment associated with the myopic axial elongation. Previous histomorphometric investigations revealed that the axial myopic elongation leads to an enlargement of the globes mainly in its posterior segment, starting mostly at or behind the equator and being more pronounced the closer to the posterior pole [20]. The association between longer disc-fovea distance and longer axial length may also explain the association between a longer disc-fovea distance and larger parapapillary beta/gamma zone. Parapapillary beta/gamma zone was defined in this study by visible sclera and visible large choroidal vessels [10,21,22]. This definition of beta/gamma zone is based on the assessment of photographs. Recent clinical studies applying OCT imaging techniques and histologic investigations have shown that the “former” beta zone can further be differentiated into an OCT defined beta zone characterized by the presence of Bruch´s membrane and absence of retinal pigment epithelium, and into a gamma zone, which is located between beta zone and the optic disc border and which is characterized by an absence of Bruch´s membrane [23,24]. The (new) beta zone is typically associated with glaucomatous optic nerve damage, and the gamma zone is associated with axial myopia. We therefore excluded glaucomatous eyes in the second part of the statistical analysis and assumed that the “former” beta zone in the remaining non-glaucomatous eyes was mostly gamma zone. If this gamma zone and the disc radius were subtracted from the disc-fovea distance, the remaining segment was the part in which Bruch´s membrane was present. It was called BMO-fovea distance since Bruch´s membrane opening included the intrapapillary optic nerve head region plus gamma zone. Interestingly, the BMO-fovea distance was not significantly related with axial length in eyes with an axial length of equal to or larger than 23.5 mm, roughly representing emmetropic eyes and axially myopic eyes. It suggests that the myopic axial elongation was not associated with a stretching and lengthening of Bruch´s membrane in the macular region but that the axial elongation associated increase in the disc-fovea distance was due to the development or enlargement of parapapillary gamma zone. In agreement with this observation, a recent histomorphometric study showed that Bruch´s membrane thickness was not significantly associated with axial length. Axially elongated eyes showed a markedly reduced thickness of the sclera and choroid, while Bruch´s membrane thickness did not differ between normal eyes and eyes with axial elongation [25].

The finding that the BMO-fovea distance as a surrogate for the length of the macular Bruch´s membrane (as measured from the posterior pole to nasal end of Bruch´s membrane in direction to the optic disc) was mostly independent of axial length suggests that the axial elongation associated increase in the fovea-disc distance led to the appearance or enlargement of a papillary gamma zone. This finding makes one infer that the distance between the retinal photoreceptors in the macular and foveal region was not markedly dependent on axial length as long as highly myopic eyes with secondary macular Bruch´s membrane defects are excluded. Correspondingly, a recent multivariate analysis revealed that within non-highly myopic eyes (i.e. eyes with an axial length of less than 26 mm), better best corrected visual acuity was significantly associated with thicker subfoveal choroid (*P*<0.001) in general and a subfoveal choroid thicker than 30 μm (*P*<0.001) in particular, while it was not significantly with axial length, after adjusting for younger age (*P*<0.001), higher level of education (*P*<0.001), taller body stature (*P*<0.001), higher body mass index (*P* = 0.005), and absence of major ocular diseases such as glaucoma [26].

The finding that the length of Bruch´s membrane as measured from the posterior pole to the end of parapapillary beta/gamma zone and the finding of an increase in the fovea-disc border distance by an enlargement of parapapillary gamma zone may imply that Bruch´s membrane is not firmly fixed on the underlying sclera through the choroid but may slightly slide. Sliding of Bruch´s membrane has already been discussed in a previous study in which parapapillary gamma zone markedly decreased in size after a profound reduction in intraocular pressure had occurred [27]. As an analogy, a sliding of the retinal pigment epithelium on Bruch´s membrane has recently been proposed to be involved in the etiology of parapapillary atrophy in glaucoma [28].

The association between longer disc-fovea distance and larger optic nerve head may be due to at least two reasons. First, myopic, axially elongated eyes can have, due to the enlargement of the posterior segment, a secondarily enlarged optic disc [10]. Second, non-highly myopic eyes with primarily large discs have overall large ocular dimensions with primarily large corneas, and long horizontal and vertical globe diameters [29,30].

It has remained unclear why a longer optic disc-fovea distance was significantly associated with a lower degree of cortical cataract (*P* = 0.002), and lower prevalence of age-related macular degeneration (*P* = 0.001) (Table 1). The relationship with a lower frequency of age-related macular degeneration might have been due to the association between longer axial length and lower prevalence of age-related macular degeneration as it had been shown in the Beijing Eye Study and other population-based investigations [31–33]. The associations of disc-fovea distance with arterial blood pressure and blood concentration of lipoproteins and of glucose were statistically significant only in the univariate analysis, while the associations lost their significance in the multivariate analysis. One may therefore assume that confounding factors were the cause for the associations found in the univariate analysis.

The difference between the disc diameter estimation based on the disc-fovea distance and the direct disc diameter measurement suggested that the clinical applicability of using the ratio of disc-fovea distance to disc diameter as relative size to estimate the size of structures at the posterior pole was limited. It relates to a study by Mok and colleagues who measured the (horizontal) disc-to-fovea distance and optic disc diameter in 88 normal subjects [4]. In contrast to our study, Mok and coworkers did not find significant differences in the disc-fovea distance among three groups of eyes with different optic disc size. Subsequently, Mok and colleagues reported that the ratio of the disc-fovea distance divided by the disc diameter was significantly lower in individuals with physiological macrodiscs (ratio: 1.91 ± 0.07) than in normal individuals (2.54 ± 0.13) and in glaucomatous patients (2.50 ± 0.15). Mok and coworkers concluded that the ratio of disc-fovea distance divided by the disc diameter could be used as a relative size unit for the estimation of the optic disc size.

Interestingly, longer disc-fovea distance was associated with deeper anterior chamber depth in the univariate analysis (*P*<0.001; r: 0.11), while in multivariate analysis, longer disc-fovea distance was correlated with more shallow chamber depth (*P*<0.001; beta: -0.18) (Table 1). This change in the direction of the association may be explained by the influence of the other parameters in the multivariate analysis. If anterior chamber depth was dropped from the multivariate analysis, the parameter for lens thickness was no longer significantly associated with disc-fovea distance (*P* = 0.34), while all other parameters remained to be significantly correlated with the disc-fovea distance as described above.

Potential limitations of our study should be mentioned. First, our study had a lower age limit of 50 years so that the findings of our study could not be transferred on younger individuals. Second, readable fundus photographs and axial length measurements were available for 81.8% of the participants, and the original participation rate of all eligible subjects was 78.8%. These figures may, however, be sufficient to allow conclusions on normative values such as the disc-fovea distance and its associations. Third, the present study included Chinese individuals. Since ocular dimensions may differ between ethnicities, the measurements obtained in our study population may not directly be transferred onto other populations. Fourth, we assumed that the ophthalmoscopical beta/gamma zone in non-glaucomatous eyes predominantly represented gamma zone. Without having assessed OCT images in a systematic manner for the study participants, this assumption could not be proven. Previous histological studies and clinical investigations had shown however, that beta zone was usually associated with glaucoma and that gamma zone was usually associated with axial myopia [23,24]. Since we excluded eyes with glaucoma in our study on the parapapillary beta/gamma zone, the parapapillary beta/gamma zone in our specific study population may predominantly have been presented gamma zone. Fifth, the assessment of a distance on fundus photographs is a two-dimensional examination although the real distance between the disc center and the fovea is longer than the chord length measured and presented in this study.

In conclusion, the optic disc center-fovea distance (mean: 4.76 ± 0.3 4 mm) increased with longer axial length, larger parapapillary alpha zone, larger parapapillary beta/gamma zone and larger optic disc area. Since the Bruch´s membrane opening-fovea distance was not related with axial length in emmetropic or myopic eyes (corresponding to an axial length of ≥23.5 mm), the axial elongation associated increase in the disc-fovea distance was markedly due to an enlargement of parapapillary beta/gamma zone while Bruch´s membrane itself was presumably not stretched or lengthened. This finding may be of interest for the process of emmetropization and myopization.

## References

[1] Wakakura M, Alvarez E. A simple clinical method of assessing patients with optic nerve hypoplasia. The disc-fovea distance to disc diameter ratio (DM/DD). Acta Ophthalmol (Copenh). 1987;65:612–617. 3425270 10.1111/j.1755-3768.1987.tb07051.x

[2] Alvarez E, Wakakura M, Khan Z, Dutton GN. The disc-fovea distance to disc diameter ratio: a new test for confirming optic nerve hypoplasia in young children. J Pediatr Ophthalmol Strabismus. 1988;25:151–154. 3397860 10.3928/0191-3913-19880501-11

[3] Barr DB, Weir CR, Purdie AT. An appraisal of the disc-macula distance to disc diameter ratio in the assessment of optic disc size. Ophthalmic Physiol Opt. 1999;19:365–375. 10768018 10.1046/j.1475-1313.1999.00463.x

[4] Mok KH, Lee VW. Disc-to-macula distance to disc-diameter ratio for optic disc size estimation. J Glaucoma. 2002;11:392–395. 12362077 10.1097/00061198-200210000-00004

[5] Rohrschneider K. Determination of the location of the fovea on the fundus. Invest Ophthalmol Vis Sci. 2004;45:3257–3258. 15326148 10.1167/iovs.03-1157

[6] De Silva DJ, Cocker KD, Lau G, Clay ST, Fielder AR, Moseley MJ. Optic disk size and optic disk-to-fovea distance in preterm and full-term infants. Invest Ophthalmol Vis Sci. 2006;47:4683–4686. 17065474 10.1167/iovs.06-0152

[7] van de Put MA, Nayebi F, Croonen D, Nolte IM, Japing WJ, Hooymans JM, et al Design and validation of a method to determine the position of the fovea by using the nerve-head to fovea distance of the fellow eye. PLoS One. 2013;8:e62518 10.1371/journal.pone.0062518 23667483 PMC3646827

[8] Lee M, Jin H, Ahn J. Relationship between disc margin to fovea distance and central visual field defect in normal tension glaucoma. Graefes Arch Clin Exp Ophthalmol. 2014;252:307–314. 10.1007/s00417-013-2513-2 24263528

[9] Knaapi L, Lehtonen T, Vesti E, Leinonen MT. Determining the size of retinal features in prematurely born children by fundus photography. Acta Ophthalmol. 2015;93:339–341. 10.1111/aos.12554 25270671

[10] Xu L, Li Y, Wang S, Wang Y, Wang Y, Jonas JB. Characteristics of highly myopic eyes. The Beijing Eye Study. Ophthalmology. 2007;114:121–126. 17070594 10.1016/j.ophtha.2006.05.071

[11] Wang YX, Xu L, Yang H, Jonas JB. Prevalence of glaucoma in North China: The Beijing Eye Study. Am J Ophthalmol. 2010;150:917–924. 10.1016/j.ajo.2010.06.037 20970107

[12] Wei WB, Xu L, Jonas JB, Shao L, Du KF, Wang S, et al Subfoveal choroidal thickness: the Beijing Eye Study. Ophthalmology. 2013;120:175–180. 10.1016/j.ophtha.2012.07.048 23009895

[13] Shao L, Xu L, Zhang JS, You QS, Chen CX, Wang YX, et al Subfoveal choroidal thickness and cataract. The Beijing Eye Study 2011. Invest Ophthalmol Vis Sci. 2015;56:810–815. 10.1167/iovs.14-15736 25604689

[14] Xu J, Xu L, Wang YX, You QS, Jonas JB, Wei WB. Ten-year cumulative incidenceof diabetic retinopathy. The Beijing Eye Study 2001/2011. PLoS One. 2014;9:e111320 10.1371/journal.pone.0111320 25347072 PMC4210166

[15] Xu L, Wang Y, Yang H, Jonas JB. Differences in parapapillary atrophy between glaucomatous and normal eyes: the Beijing Eye Study. Am J Ophthalmol. 2007;144:541–546. 17651676 10.1016/j.ajo.2007.05.038

[16] Jonas JB, Xu L, Zhang L, Wang Y, Wang Y. Optic disk size in chronic glaucoma. The Beijing Eye Study. Am J Ophthalmol. 2006;142:168–170. 16815273 10.1016/j.ajo.2006.01.068

[17] Littmann H. [Determination of the real size of an object on the fundus of the living eye]. Klin Monbl Augenheilkd. 1982;180:286–289. 7087358 10.1055/s-2008-1055068

[18] Bennett AG, Rudnicka AR, Edgar DF. Improvements on Littmann's method of determining the size of retinal features by fundus photography. Graefes Arch Clin Exp Ophthalmol. 1994;232:361–367. 8082844 10.1007/BF00175988

[19] Jonas JB, Gusek GC, Guggenmoos-Holzmann I, Naumann GO. Size of the optic nerve scleral canal and comparison with intravital determination of optic disc dimensions. Graefes Arch Clin Exp Ophthalmol. 1988;226:213–215. 3402742 10.1007/BF02181183

[20] Vurgese S, Panda-Jonas S, Jonas JB. Scleral thickness in human eyes. PLoS One. 2012;7:e29692 10.1371/journal.pone.0029692 22238635 PMC3253100

[21] Jonas JB, Fernández MC, Naumann GO. Parapapillary atrophy and retinal vessel diameter in nonglaucomatous optic nerve damage. Invest Ophthalmol Vis Sci. 1991;32:2942–2947. 1917397

[22] Jonas JB, Budde WM. Diagnosis and pathogenesis of glaucomatous optic neuropathy: morphological aspects. Prog Retin Eye Res. 2000;19:1–40. 10614679 10.1016/s1350-9462(99)00002-6

[23] Jonas JB, Jonas SB, Jonas RA, Holbach L, Dai Y, Sun X, et al Parapapillary atrophy: Histological gamma zone and delta zone. PLoS One. 2012;7:e47237 10.1371/journal.pone.0047237 23094040 PMC3475708

[24] Dai Y, Jonas JB, Huang H, Wang M, Sun X. Microstructure of parapapillary atrophy: Beta zone and gamma zone. Invest Ophthalmol Vis Sci. 2013;54:2013–2018. 10.1167/iovs.12-11255 23462744

[25] Jonas JB, Holbach L, Panda-Jonas S. Bruch´s membrane thickness in high myopia. Acta Ophthalmol. 2014;92:e470–474. 10.1111/aos.12372 24612938

[26] Shao L, Xu L, Wei WB, Chen CX, Du KF, Li XP, et al Visual acuity and subfoveal choroidal thickness. The Beijing Eye Study. Am J Ophthalmol. 2014;158:702–709. 10.1016/j.ajo.2014.05.023 24878308

[27] Panda-Jonas S, Xu L, Yang H, Wang YX, Jonas SB, Jonas JB. Optic disc morphology in young patients after antiglaucomatous filtering surgery. Acta Ophthalmol. 2014;92:59–64. 10.1111/j.1755-3768.2012.02570.x 23356397

[28] Wang YX, Jiang R, Wang NL, Xu L, Jonas JB. Acute peripapillary retinal pigment epithelium changes associated with acute intraocular pressure elevation. Ophthalmology 2015 7 15 pii: S0161-6420(15)00551-5. 10.1016/j.ophtha.2015.06.005 [Epub ahead of print]. 26189187

[29] Jonas JB, Zäch FM, Gusek GC, Naumann GO. Pseudoglaucomatous physiologic large cups. Am J Ophthalmol. 1989;107:137–144. 2913807 10.1016/0002-9394(89)90212-2

[30] Papastathopoulos KI, Jonas JB, Panda-Jonas S. Large optic discs in large eyes, small optic discs in small eyes. Exp Eye Res. 1995;60:459–461. 7789425 10.1016/s0014-4835(05)80102-2

[31] Xu L, Li Y, Zheng Y, Jonas JB. Associated factors for age related maculopathy in the adult population in China: the Beijing eye study. Br J Ophthalmol. 2006;.90:1087–1090. 16774957 10.1136/bjo.2006.096123 PMC1857376

[32] Jonas JB, Nangia V, Kulkarni M, Gupta R, Khare A. Associations of early age-related macular degeneration with ocular and general parameters. The Central India Eyes and Medical Study. Acta Ophthalmol. 2012;90:e185–191. 10.1111/j.1755-3768.2011.02316.x 22269029

[33] Pan CW, Ikram MK, Cheung CY, Choi HW, Cheung CM, Jonas JB, et al Refractive errors and age-related macular degeneration: a systematic review and meta-analysis. Ophthalmology 2013;120:2058–2065. 10.1016/j.ophtha.2013.03.028 23706699

